# Supplementary material for: Contrast-enhanced ultrasound measurement of pancreatic blood flow dynamics predicts type 1 diabetes progression in preclinical models
Source: Nat Commun. 2018 May 1;9:1742. doi: 10.1038/s41467-018-03953-y (PMC5931596; doi:10.1038/s41467-018-03953-y)
Supplement: Supplementary file 1 — Supplementary Information [file 41467_2018_3953_MOESM1_ESM.pdf]

Supplementary Information

Contrast-enhanced ultrasound measurement of pancreatic blood flow dynamics predicts type1 diabetes progression in preclinical models

St Clair et al.

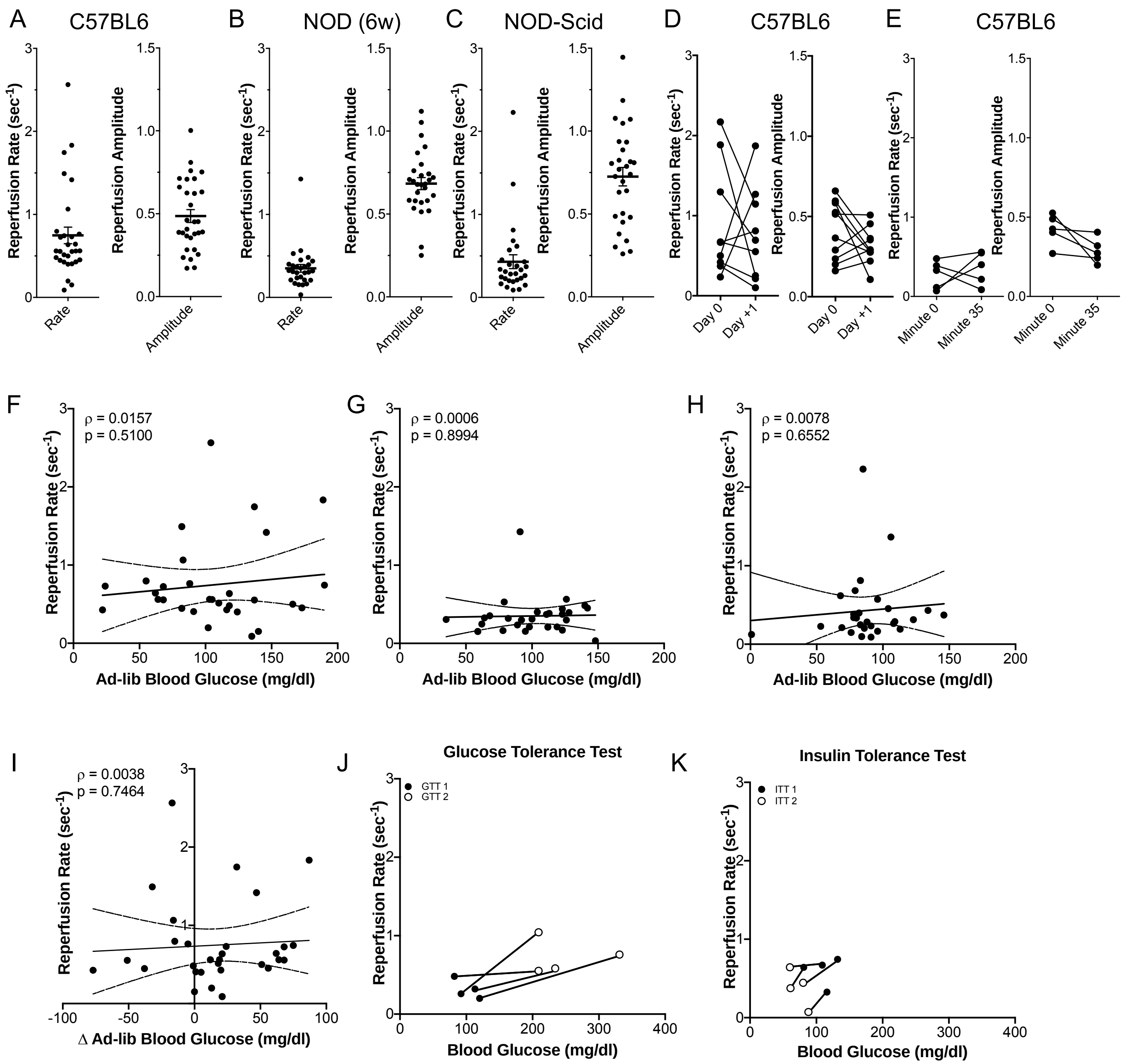

Supplementary Figure 1: Comparisons of reperfusion rate and amplitude across study animal genotypes. (A) Reperfusion rate and amplitude for all C57BL6 females used in this study. (B) as in A for NOD females. (C) as in A for NOD-Scid. (D). Reperfusion rates and amplitudes performed in successive measures, 1 day apart in C57BL6 mice. (E) As in D 35 minutes apart in C57BL6 mice. (F) Correlation (solid line linear regression, dashed lines 95% confidence intervals) between reperfusion rate and blood glucose at time of scan while under isoflurane anesthesia for all C57BL6 mice used in this study. (G) as in F for NOD females. (H) as in F for NOD-Scid. (I) Correlation between reperfusion rate and change in blood glucose from before anesthesia application to after the scan while under isoflurane anesthesia for all C57BL6 mice used in this study. (J) Change in reperfusion rate and change in blood glucose for mice receiving a scan before and during a Glucose Tolerance Test (GTT). (K) As in J for mice receiving a scan before and during an Insulin Tolerance Test (ITT). Data presented represents following numbers of mice: n=30 (A), n=28(B), n=28 (C), n=9 (D), n=5 (E), n=30 (F), n=28 (G), n=28 (H), n=30 (I), n=4 (J), n=4 (K).

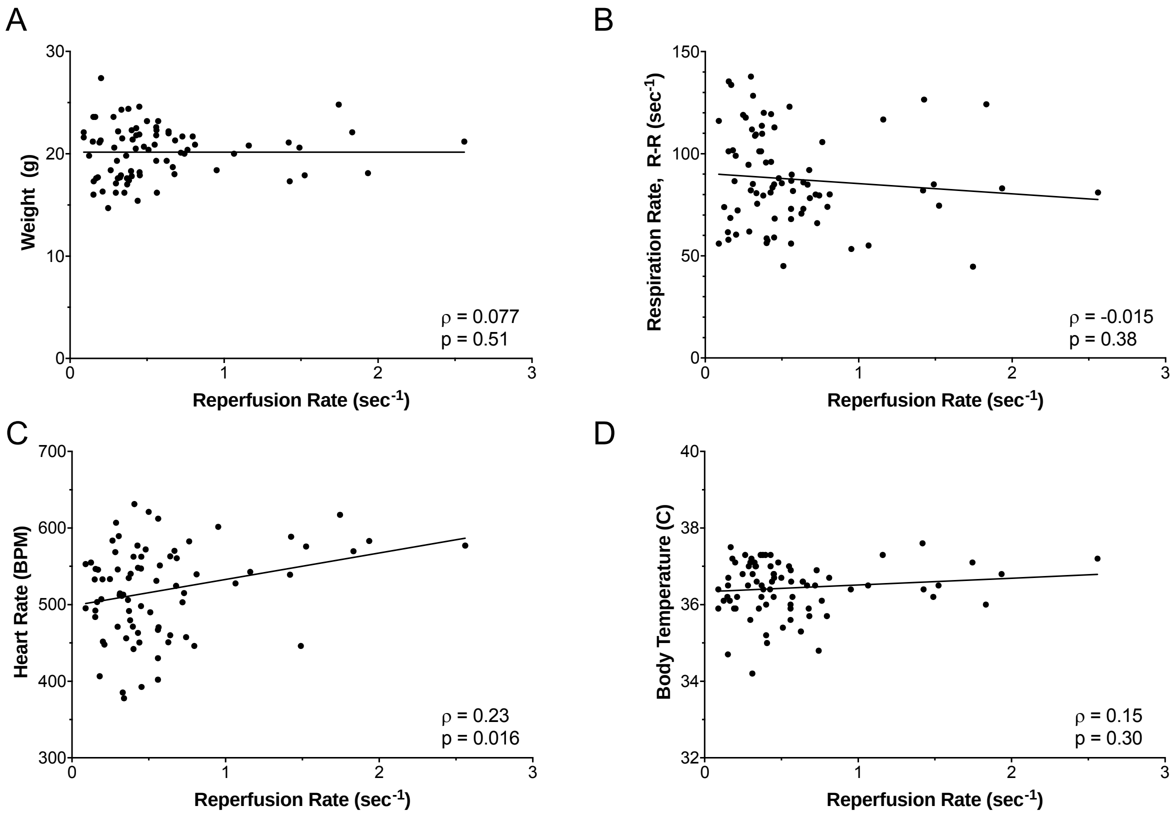

Supplementary Figure 2: Compilation of C57BL6 and NOD physiological measures and reperfusion parameters measured at time of CEUS scan. (A) Correlations of pancreas reperfusion rate in all C57BL6 and NOD animals compared to the animal's weight at the time of scan. (B) As in A for respiration rate. (C) As in A for heart rate. (D) As in A for body temperature. Correlation coefficients (ρ) and statistical significance (p) were determined by Pearsons correlation analysis with Bonferroni correction for multiple comparisons, and are as indicated. Data presented represents n=79 mice.

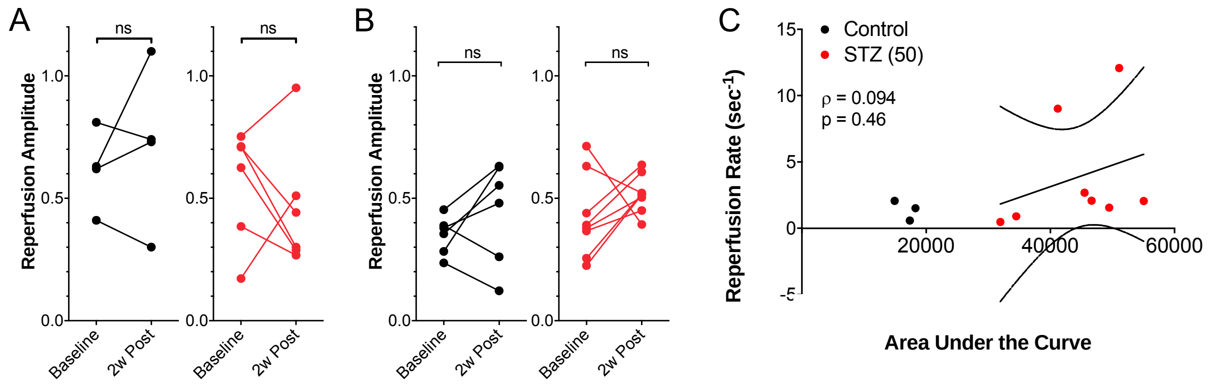

Supplementary Figure 3: STZ-treated mice additional reperfusion parameters and comparisons. (A) Reperfusion amplitudes in control and STZ treated mice (70mg/kg) mice showing changes in individual mice. (B) As in A for control and STZ treated mice (50mg/kg) mice. (C) Correlation of reperfusion rate and area under the curve during a glucose tolerance test for control and STZ treated mice (50mg/kg) mice. Correlation coefficients (ρ) and statistical significance (p) were determined by Pearsons correlation analysis among STZ treated mice. ns $p>0.1$ comparing groups indicated (Paired t-test). Data in A represents n=6 STZ-treated and n=4 control mice; data in B represents n=8 STZ-treated and n=6 control mice; data in C represents n=8 STZ-treated and n=3 control mice.

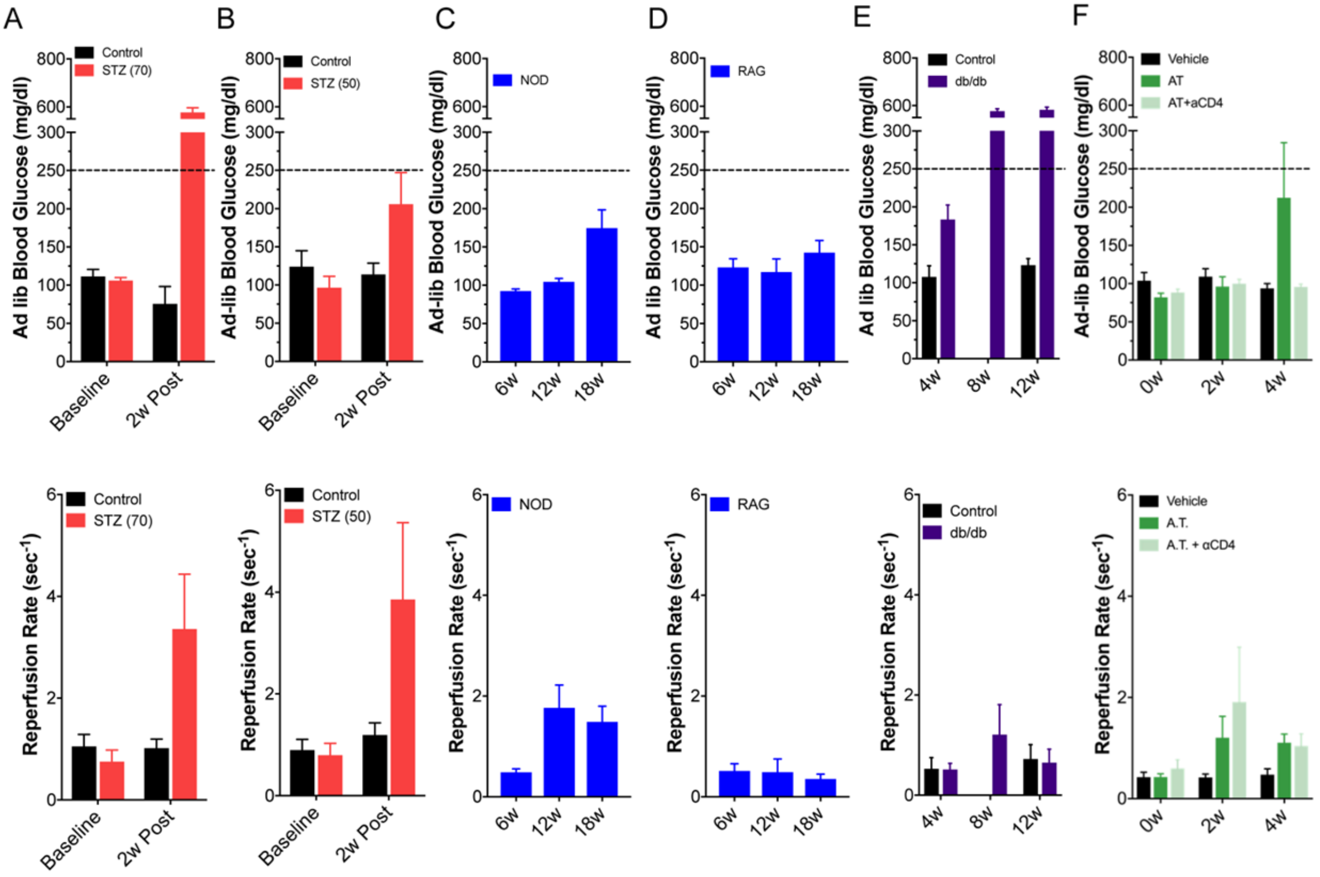

Supplementary Figure 4: Summary of blood glucose levels and reperfusion rates measured during disease progression in the models examined. (A) Mean (±s.e.m.) ad lib blood glucose at time of scan (above) and mean (±s.e.m.) reperfusion rate measured (below) for STZ-treated mice (70mg/kg) and vehicle controls prior to treatment (*Baseline*) and 2 weeks post treatment (*2w Post*). Dashed line indicates criteria used to indicate for 'diabetes' in NOD mice. (B) As in A for lower dose STZ-treated mice (50mg/kg) and vehicle controls. (C) As in A for NOD mice at 6, 12, 18 weeks of age. (D) As in A for NOD-RAG1$^{-/-}$ mice at 6, 12, 18 weeks age. (E) As in A for d/db mice and C57BLKS control mice at 4, 8, 12 weeks of age. (F) As in A for A.T. mice, either untreated or antiCD4 treated, and vehicle treated NOD-scid controls. Error bars represent s.e.m. Data in A represents n=6 STZ-treated (n=9 at baseline) and n=5 control mice (n=8 at baseline); data in B represents n=8 STZ-treated and n=6 control mice; data in C represents n=35 NOD mice (n=24 at 18w); data in D represents n=6 NOD-RAG1$^{-/-}$ mice (n=5 at 12, 18w); data in E represents n=8 db/db mice and 4 C57BLKS mice; data in F represents n=6 AT mice, n=12 AT+αCD4 mice and n=6 control mice.

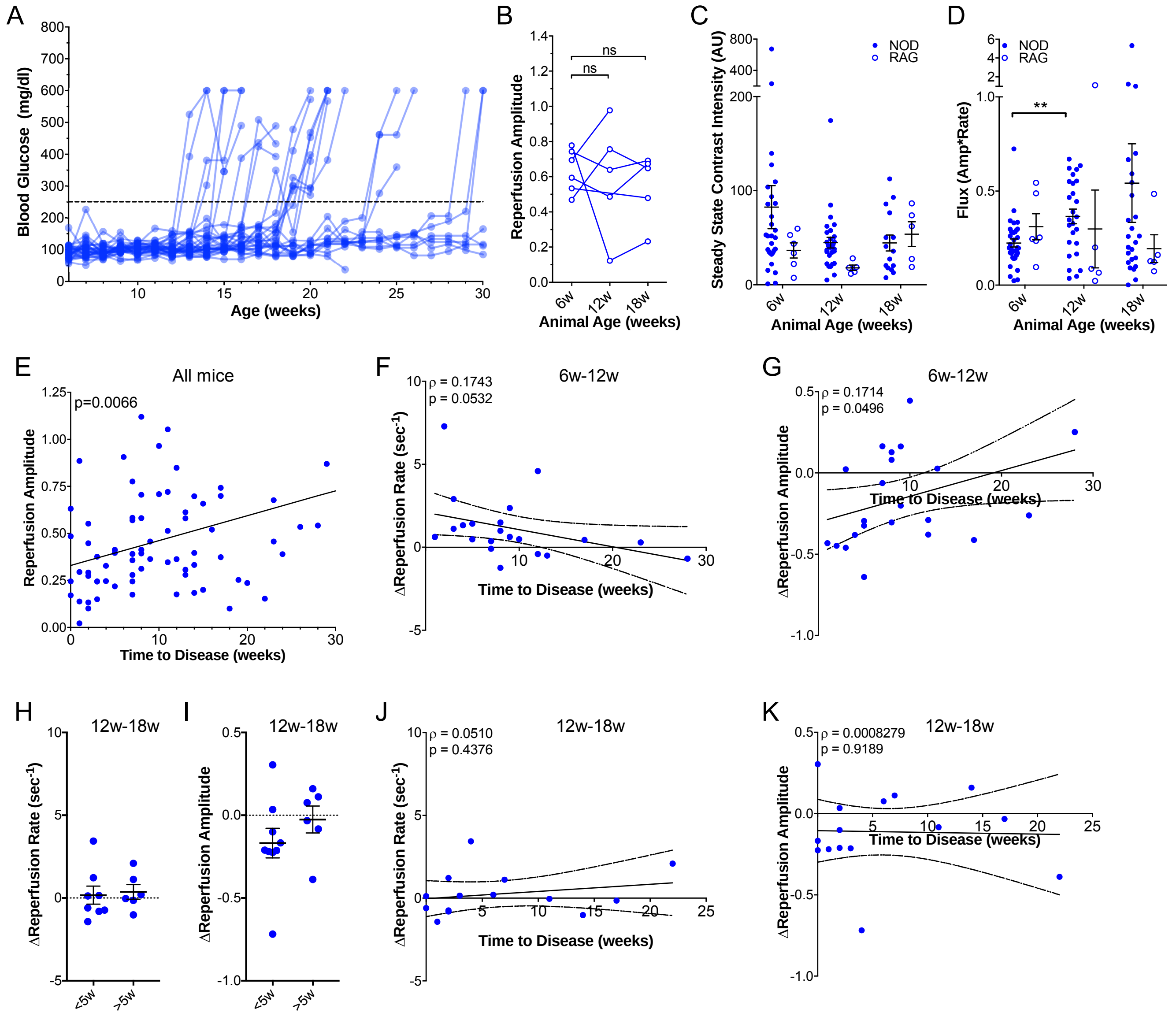

Supplementary Figure 5: NOD mice additional reperfusion parameters and comparisons. (A) Ad libidum blood glucose measurements in all female NOD mice over time used in this study. (B) Reperfusion amplitudes in NOD-RAG1$^{-/-}$ mice showing changes in individual mice between 6 and 18 weeks age. (C) Steady state contrast intensity measured in NOD (solid blue) and NOD-RAG1$^{-/-}$ (open blue) mice at 6, 12 and 18 week ages indicated.(D) Calculated contrast flux (amplitude * rate) in NOD (solid blue) and NOD-RAG1$^{-/-}$ (open blue)mice at 6, 12 and 18 week ages indicated. (E) Correlations of reperfusion amplitude with time to diabetes from CEUS scan, in weeks. (F) Correlations of change in reperfusion rate from 6 to 12 weeks with time to diabetes from CEUS scan, in weeks. Trend line indicates linear regression with 95% confidence intervals. (G) as in F for change in reperfusion amplitude from 6 to 12 weeks. (H) Average change in reperfusion rate from 12 to 18 weeks, averaged over animals (in J) that progressed to disease <5 weeks or >5 weeks from CEUS scan, (I) As in H for change in reperfusion amplitude form 12 to 18 weeks. (J) Correlations of change in reperfusion rate from 12 to 18 weeks with time to diabetes from CEUS scan, in weeks. Trend line indicates linear regression with 95% confidence intervals. (K) as in F for change in reperfusion amplitude from 12 to 18 weeks. Data presented represents following numbers of mice: n=31 (A); n=6 (B); n=29 NOD, n=5 NOD-RAG1$^{-/-}$; (C,D); n=71 scans, 27 mice (E); n=27 (F,G) n=15 (H-K). *p<0.05, **p<0.01 as determined by paired t-tests compared to 6 week baseline scan. A mixed-effects model was used to assess the statistical significance and generate the regression in E.

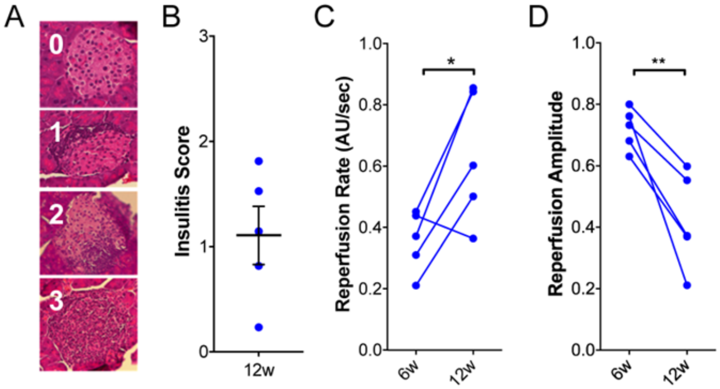

Supplementary Figure 6: Insulitis in NOD animals. (A) Representative images of H&E stained pancreas sections from a 12 week old female NOD mouse: 0: no infiltration, 1:peri-islet infiltration, 2: <50% of islet infiltrated, 3: >50% of islet infiltrated. (B) Mean insulitis score for each NOD animal used in this analysis, indicated mild to moderate insulitis at 12 weeks of age. A minimum of 60 islets were blindly analyzed for each animal. (C) Changes in reperfusion rate from 6 to 12 weeks of age in individual female NOD mice used for insulitis scoring. (D) Changes in reperfusion amplitude, as in C. *$p<0.05$ in as assessed via paired t-test. Data in B-D represents n=5 NOD mice.

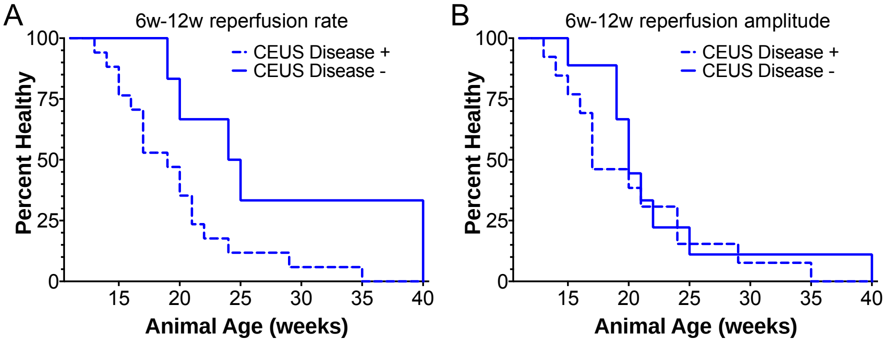

C

Metric	Comparison	p (...)
6w-12w Δrate	Disease+ vs Disease-	p<0.0001
6w-12w Δamp	Disease+ vs Disease-	p=0.003
12w rate	Disease+ vs Disease-	p<0.0001
12w amp	Disease+ vs Disease-	p=0.23

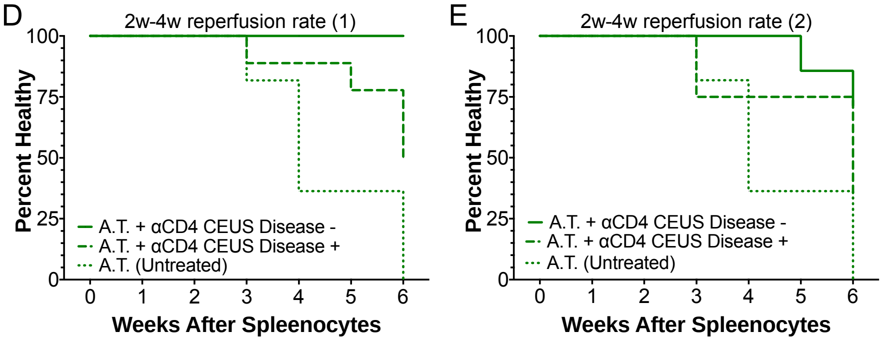

F

Metric	Comparison	p (...)
2w-4w Δrate (1)	Disease+ vs Disease-	p=0.09
2w-4w Δrate (1)	Disease+ vs A.T.	p=0.06
2w-4w Δrate (1)	Disease- vs A.T.	p=0.06
2w-4w Δrate (2)	Disease+ vs Disease-	p=0.05
2w-4w Δrate (2)	Disease+ vs A.T.	p=0.1
2w-4w Δrate (2)	Disease- vs A.T.	p=0.05
4w rate	Disease+ vs Disease-	p=0.23
4w rate	Disease+ vs A.T.	p=0.06
4w rate	Disease- vs A.T.	p=0.05

Supplementary Figure 7: Prediction of diabetes progression by measurements of reperfusion rate or amplitude. (A) Survival curves indicating diabetes development of NOD mice that are predicted to develop diabetes rapidly (Disease +) or develop diabetes slowly (Disease -) based on measurements of the change in reperfusion rate between 6 and 12 weeks. Prediction was based on whether the change in rate was greater than (Disease +) or less than (Disease -) a threshold rate that optimally separated the change in reperfusion rate between NOD and NOD-RAG1$^{-/-}$ mice. (B) Survival curves indicating diabetes development as in A considering change in reperfusion amplitude between 6 and 12 weeks. (C) Summary comparing survival curves in A,B and those assessed using other indicated metrics, as determined by Mantel-Cox test. (D) Survival curves indicating diabetes development of antiCD4-treated A.T. mice that are predicted to not respond to antiCD4 treatment and develop diabetes (non-responder, Disease +) or that are predicted respond to antiCD4 treatment and not develop diabetes (responder, Disease -), based on measurements of the change in reperfusion rate between 2 and 4 weeks after splenocyte transfer. Prediction was based on whether the change in rate was greater than (Disease +) or less than (Disease -) a threshold rate that optimally separated the change in reperfusion rate between untreated AT and control mice. (E) as in D using a second optimal separation threshold. (F) Summary comparing survival curves in D,E and those assessed using other indicated metrics, as determined by Mantel-Cox test. Data in A-C generated from n=31 NOD mice; data in D-F generated from n=12 AT+αCD4 mice and n=6 untreated AT mice.

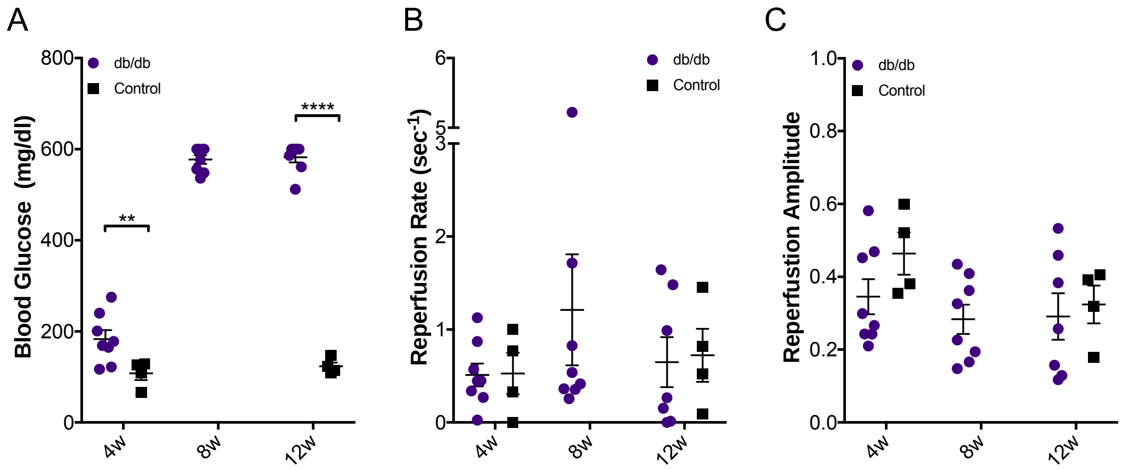

Supplementary Figure 8: CEUS shows little change in pancreas blood flow dynamics associated with the progression of diabetes in db/db mice. (A) Ad-libidum blood glucose concentrations of male db/db mice and C57BLKS control animals at the ages indicated. (B) Reperfusion rate measured in mice in A. (C) Reperfusion amplitude measured in mice in A. *p<0.05, **p<0.01, ***p<0.001, ****p<0.0001 comparing groups indicated (ANOVA data in A-C). Data in A-C represents n=8 db/db mice and 4 C57BLKS mice

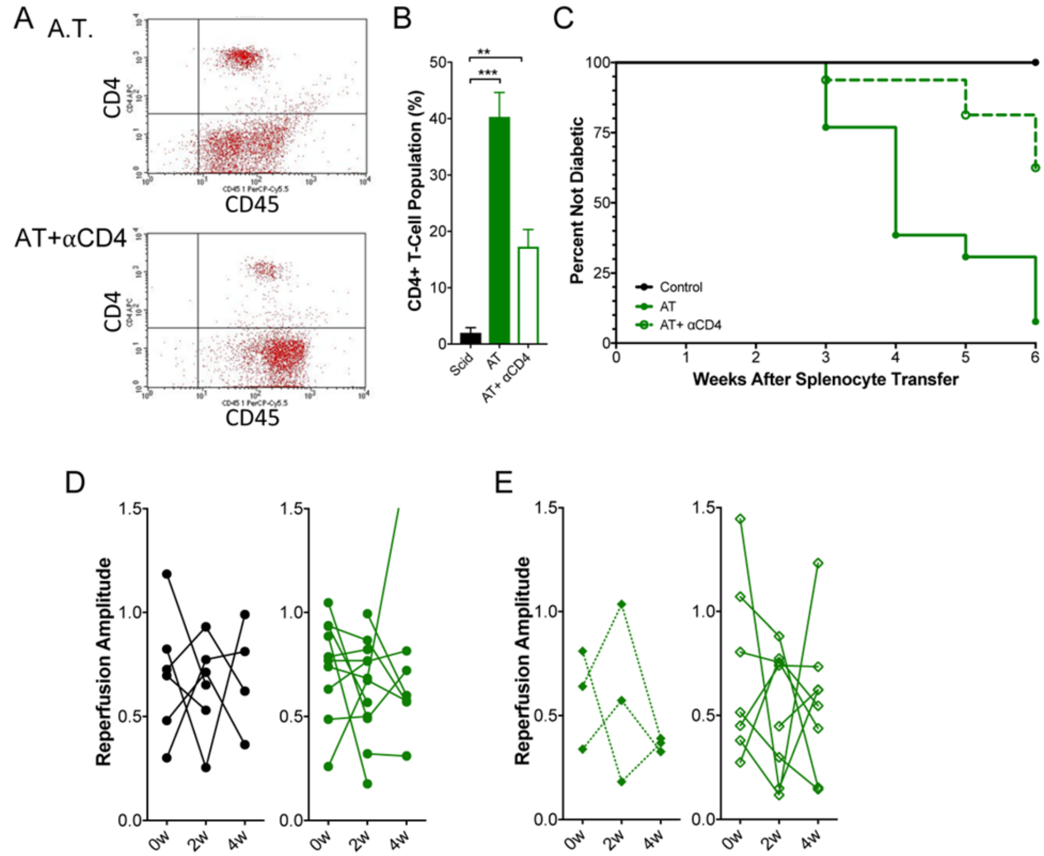

Supplementary Figure 9: Transfer of diabetogenic splenocytes results in CD4 positive T-cell populations that were diminished by anti-CD4 immunotherapy in vivo. (A) Representative flow cytometry plots of CD45.1 positive (x-axis) and CD4 positive (y-axis) from transferred (AT) and transferred with anti-CD4 treatment (AT+αCD4) from whole blood samples. (B) Quantification of CD4-positive T-cell population as a percent of CD45.1 expressing cells (Scid n=4, AT n=4, AT+αCD4 n=8). (C) Survival curves indicating diabetes development for control (black), AT (green), and AT+αCD4 (open hashed). (D) Reperfusion amplitudes in control (left) and AT mice (right) showing changes in individual mice between 0 and 4 weeks post splenocyte transfer. (E) As in D for antiCD4 non-responder AT mice (left) and antiCD4 responder AT mice (right). Error bars represent s.e.m. Survival curves significantly different from another, $p<0.0001$, as determined by Mantel-Cox test. $^{**}p<0.01$, $^{***}p<0.001$ (ANOVA for data in B). Data in B represents n=4 mice (n=8 AT+αCD4); data in C generated from n=4 control, n=6 AT and n=12 AT+antiCD4 mice; data in D represents n=11 AT mice and n=6 control mice, data in E represents n=9 responder mice and 3 non-responder mice. Data in B represents the top-right quadrant population ($CD45^+$,$CD4^+$) as a % of the top-right plus bottom-right quadrants population ($CD45^+$) shown in A.
